# Harnessing the Potential of miRNAs in Malaria Diagnostic and Prevention

**DOI:** 10.3389/fcimb.2021.793954

**Published:** 2021-12-16

**Authors:** Himanshu Gupta, Samuel C. Wassmer

**Affiliations:** Department of Infection Biology, London School of Hygiene and Tropical Medicine, London, United Kingdom

**Keywords:** miRNAs, malaria, *Plasmodium*, *Anopheles*, biomarkers, diagnosis

## Abstract

Despite encouraging progress over the past decade, malaria remains a major global health challenge. Its severe form accounts for the majority of malaria-related deaths, and early diagnosis is key for a positive outcome. However, this is hindered by the non-specific symptoms caused by malaria, which often overlap with those of other viral, bacterial and parasitic infections. In addition, current tools are unable to detect the nature and degree of vital organ dysfunction associated with severe malaria, as complications develop silently until the effective treatment window is closed. It is therefore crucial to identify cheap and reliable early biomarkers of this wide-spectrum disease. microRNAs (miRNAs), a class of small non-coding RNAs, are rapidly released into the blood circulation upon physiological changes, including infection and organ damage. The present review details our current knowledge of miRNAs as biomarkers of specific organ dysfunction in patients with malaria, and both promising candidates identified by pre-clinical models and important knowledge gaps are highlighted for future evaluation in humans. miRNAs associated with infected vectors are also described, with a view to expandind this rapidly growing field of research to malaria transmission and surveillance.

## Highlights

- miRNAs have recently emerged as essential regulators of immunity against *Plasmodium* parasites both in mosquito vectors and human hosts- Specific miRNAs have been associated with severe malaria and its associated life-threatening complications- miRNAs present promising diagnostic/prognostic biomarkers of malaria disease and may also be targeted by therapeutic interventions

## Introduction

Despite encouraging control progress over the past decade, malaria remains a major global health challenge. 409,000 malaria-associated deaths were reported in 2019, and 94% of these occurred in Africa (WHO, 2020). In addition to endemic countries, fatal cases are also reported in other parts of the world due to increasing imported infections (Zoller et al., 2009; Mischlinger et al., 2020). Malaria can be caused by several *Plasmodium* species in humans, including *Plasmodium falciparum* (*Pf*), *P. vivax* (*Pv*), *P. knowlesi* (*Pk*), *P. ovale* (*Po*), and *P. malariae* (*Pm*) (Garcia, 2010). Among these, *Pf* and *Pv* are responsible for majority of cases worldwide (WHO, 2020), and *Pf* is considered the most lethal of the human malaria parasites. *Pf* cases can be classified into severe and uncomplicated malaria based on the World Health Organization (WHO) criteria (WHO, 2014). Severe *falciparum* malaria is characterized by multi-organ dysfunctions, which are triggered by the sequestration of infected erythrocytes (iEs) within the microvasculature of the host, combined with an exaggerated production of inflammatory mediators (White et al., 2013; Milner et al., 2014). In contrast, *Pv* infection has been perceived as relatively benign until recently, despite documented debilitating and potentially life-threatening complications (Gupta et al., 2015a; Gupta et al., 2016a; Anvikar et al., 2020). Severe malaria (SM) cases have also been reported in patients infected with *Pk* (Cox-Singh et al., 2010), *Po* (Kotepui et al., 2020a), and *Pm* (Kotepui et al., 2020b), although these remain anecdotal. There are distinct biochemical and morphological features across the different *Plasmodium* species, but the mechanisms underlying the development of SM are likely to be similar. They all involve inflammation caused by the release of *Plasmodium*-derived components (known as pathogen-associated molecular patterns), as well as host-derived components (or damage-associated molecular patterns) and sequestration of iEs in some specific species (Gazzinelli et al., 2014). *Plasmodium* spp. are transmitted by female *Anopheles* mosquitoes, which inject sporozoites into the subcutaneous tissue of the human host during their blood meal, triggering the host phase of the cycle (White, 2017).

The fundamental pathogenesis of SM is still poorly understood and treatments are currently precariously limited to antimalarial drugs and emergency supportive care (White et al., 2014). An early diagnosis is key for prompt and accurate treatment, resulting in positive outcomes. Unfortunately, this scenario remains rare as SM still have a fatality rate of 15-30% when patients are appropriately treated upon admission (Lucchi et al., 2011). The reasons are manifold and include *i)* late presentation to the hospital (the damage is already done); *ii*) generic symptoms such as a fever that overlaps with the presentation of viral, bacterial and parasitic infections (the infection is missed) (Rubio et al., 2016); and *iii*) out-of-criteria patients with laboratory parameters just below or above the hard WHO cut-offs for disease severity at the time of admission (the disease severity is increasing but unnoticed). For the latter category, patients treated for uncomplicated malaria (UM) have been reported to be ill enough to warrant hospitalization (Sahu et al., 2020), and could then progress to develop life-threatening SM. This suggests that complications may develop silently until it is too late (the damage is already done). A parallel study showed that UM patients with delayed treatment are highly likely to develop SM (Mousa et al., 2020). In view of these challenges, it is crucial to identify new biomarkers of early SM that can be then developed into cheap, reliable diagnostic and/or prognostic tools for clinicians to identify patients at risk.

microRNAs (miRNAs), a class of small non-coding RNAs (18-24 nt length), are rapidly released into the blood circulation upon physiological changes, including infection and organ damage (Cortez et al., 2011). They regulate gene expression endogenously at the post-transcriptional level, either through translation repression or mRNA degradation (Cortez et al., 2011). miRNAs can be secreted extracellularly as bound to lipoproteins or within cell-derived extracellular vesicles (Valadi et al., 2007; Arroyo et al., 2011; Nik Mohamed Kamal NNSB and Shahidan WNS 2020). These small molecules are highly stable and can be detected in a wide range of biological fluids, making them exceptionally promising non-invasive biomarkers (Rubio et al., 2016). Indeed, they can be potentially used to not only detect an infection, but also diagnose early-stage tissue or organ damage. This would represent a significant advantage over the standard methods currently being used to diagnose malaria, such as microscopic examination of blood smears (Fleischer, 2004), plasma antigen detection (Spencer et al., 1979), rapid diagnostic test (Moody and Chiodini, 2002) and molecular conventional and quantitative PCR assays (Snounou et al., 1993; Gupta et al., 2016b).

For these reasons, researchers using *in vitro* and mice models, as well as clinical samples have attempted to identify miRNA-based biomarkers of malarial disease in the last decade, with the hope to develop new diagnostic and prognostic tools. Remarkably, several miRNAs have also been discovered in *Pf*-infected Anopheles mosquitoes. Thus, specific profiles of miRNAs associated with different phases of the malaria life cycle have the potential to help control and elimination efforts by allowing the identification of *(i)* carrier mosquitoes, *(ii)* infected patients, symptomatic or not, and *(iii)* potential tailored treatment for the former category. The present review covers our current knowledge of miRNAs described across the malaria parasite life cycle, including both vector and host. Their potential as biomarkers of acute and chronic pathologies associated with malaria disease is discussed, as well as future research recommendations for this promising and rapidly growing field of research.

## Buzzing Around: The Vector Phase

**Figure 1:**
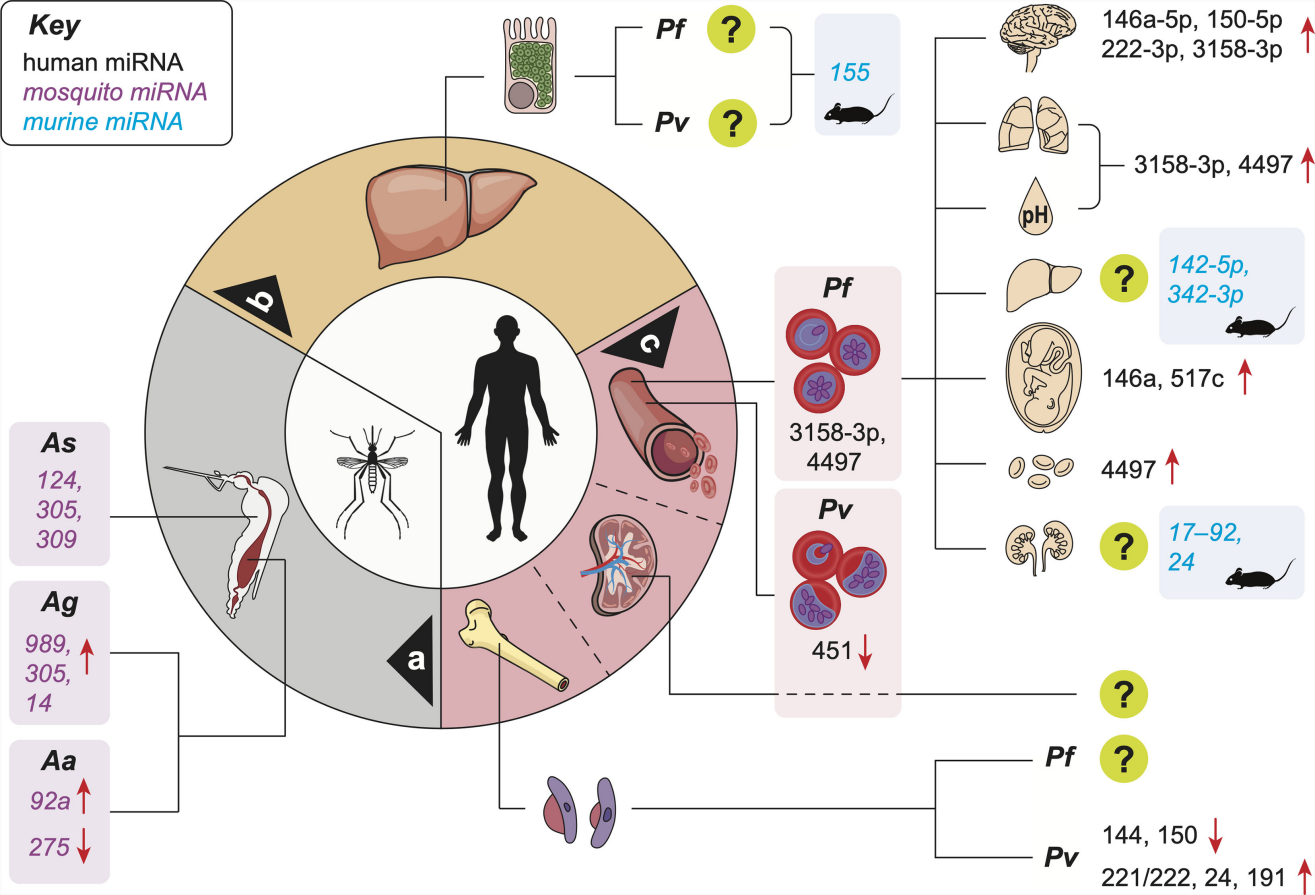
Reported miRNA expression changes in the vector and host during malaria infection. miRNA level changes in the midgut of infected mosquitoes **a**; in animal models of the infection during the silent hepatic phase **b**; and in patients during the symptomatic erythrocytic phase **c**. Organ and metabolic dysfunctions represented in the upper right quadrant are, from top to bottom: brain (cerebral malaria), lungs (ARDS), acidosis (hyperlactatemia), liver (jaundice), placenta (PAM), decreased red blood cell count (anemia), kidneys (AKI). [upregulation (**↑**); downregulation (**↓**);?: human miRNAs not identified; *Pf*, *Plasmodium falciparum*; *Pv*, *Plasmodium vivax*; *Aa*, *Anopheles anthropophagus*; *Ag*, *Anopheles gambiae*; *As*, *Anopheles stephensi*].

To complete the malaria parasite life cycle, gametocytes are ingested by female Anopheles mosquitoes from the blood of infected human hosts. Parasites then invade mosquito midgut, thereby eliciting a physiological response leading to variations in the expression and release of various miRNA. Studies of infected anopheline mosquitoes have identified several miRNAs (**Figure 1a**) that may alter mosquito immunity against *Plasmodium* infection. These miRNAs also have important roles in the development and maturation of the parasite within their vectors, and distinct expression patterns of miRNAs in specific tissues have been described (Winter et al., 2007; Mead and Tu, 2008; Jain et al., 2014; Dennison et al., 2015; Lampe et al., 2019; Dong et al., 2020). One study reported a reduction in the expression of aga-miR-34, aga-miR-1174 and aga-miR-1175 miRNAs in the midgut of *P. berghei*-infected *Anopheles gambiae* compared controls. In contrast, aga-miR-989 levels were significantly increased in the presence of the murine malaria parasite *P. berghei.* aga-miR-989 levels decreased significantly in the rest of the body, suggesting the specificity of aga-miR-989 to the midgut of infected *Anopheles gambiae*. Additionally, knocking down Dicer1 and Ago1 mRNAs enhanced the vector’s sensitivity to *Plasmodium* infection (Winter et al., 2007). Another study identified a series of differentially expressed *Anopheles gambiae* miRNAs induced by normal blood meal (miR-7, miR-92a, miR-317, and miR-N3) and infectious blood meal (miR-N3, miR-317, miR-2940, miR-N5, miR-N6 and miR-N4) (Biryukova et al., 2014). Similarly, elevated levels of aga-miR-989 and aga-miR-305 miRNAs were found in *Pf*-infected mosquito midgut tissue compared to naïve ones (**Figure 1a**). Furthermore, aga-miR-305 inhibition increased resistance to *Pf* infection (Dennison et al., 2015). Remarkably, bloodmeals have been shown to induce miR-276-5p levels in mosquito, which regulate the expression of branched-chain amino acid transferase to terminate the reproductive cycle. Inhibition of miR-276 elongated high rates of amino acid (AA) catabolism and increased female mosquito fertility, suggesting that timely termination of AA catabolism restricts both mosquito investment into reproduction and development of the transmissible sporozoite forms (Lampe et al., 2019). miRNAs (aga-miR-8, aga-miR-14, and aga-miR-305) were found to regulate mosquito immunity against parasite infection. Depletion of aga-miR-14 or aga-miR-305, but not aga-miR-8, increased mosquito resistance to both *P. berghei* and *Pf* infection by enhancing the expression of multiple immunity-related and anti-*Plasmodium* genes. This indicates a potential role for mosquito miRNAs in the development of malaria control through genetically engineered vectors (Dong et al., 2020). miRNA profiling revealed distinct expression patterns of miRNAs from early embryo to adult stages in *Anopheles stephensi*. miR-x2 was found associated with female reproduction, and constant miR-14 expression was indicative of its importance across all mosquito life stages (Mead and Tu, 2008). By applying next-generation sequencing (NGS) technology to whole vectors, differentially expressed miRNAs were reported post blood feeding (13 miRNAs) and parasite infection (16 miRNAs) in *Anopheles stephensi*. A set of miRNAs showed significant expression changes between 42h (midgut invasion) and 5 days (sporozoites release) post-infection, highlighting a stage-specific parasite influence on vector miRNAs. These miRNAs are known to target genes involved in several metabolic pathways including metabolic, redox homeostasis and protein processing machinery components. Specific miRNAs (miR-124, 305, and 309; **Figure 1a**) regulate multiple immune pathway genes (Jain et al., 2014). aan-miR-92a and aan-miR-275 levels were found upregulated and downregulated in blood-feeding and *Plasmodium* infection (**Figure 1a**), respectively, in the midgut of *Anopheles anthropophagus* compared to sugar-feeding mosquitoes (Liu et al., 2017). Collectively, these findings suggest that the expression of mosquito miRNAs changes in response to *Plasmodium* infection, and could therefore be used in molecular assays such as RT-qPCR to screen and identify parasite-carrying vectors. This would be done by comparing their miRNA levels against a baseline from uninfected vectors of the same species. In turn, such approach could help estimating metrics of exposure and transmission intensity (Tusting et al., 2014). Epidemiological and ecological studies of malaria traditionally utilize detection of *Plasmodium* sporozoites in whole mosquitoes or salivary glands by microscopy (Habluetzel et al., 1992), or serological (Wirtz et al., 1987) or molecular assays (Echeverry et al., 2017; Calzetta et al., 2018). However, these methods are time-consuming, require skill and expertise, are labour-intensive, and can over- or underestimate mosquito transmission potential (Ramirez et al., 2019). Thus, there is a need for new technologies to improve mosquito surveillance programmes, and miRNA-based assays could accurately detect infected mosquitoes in a short period of time, with the potential to inform decision-making in the fight against malaria.

## Hide: The Asymptomatic Pre-Erythrocytic Phase

Upon the bite of an infected vector, sporozoites located in its salivary glands are injected into the subcutaneous vasculature of the host. Once in the bloodstream, they travel to the liver and infect hepatocytes, initiating the silent phase of the infection, during which each sporozoite develops into hundreds of merozoites. This pre-erythrocytic phase cannot currently be identified in infected patients, but represent a pivotal window of opportunity for diagnostic and treatment, since transmission does not start until the erythrocytic stage is initiated. Identifying infected individuals at this stage could revolutionize the active surveillance programme and represent a powerful tool in malaria control. A study of the murine model of malaria infection using *P. berghei* parasites identified several miRNA candidates associated with this pre-erythrocytic phase. Indeed, murine infection with genetically attenuated parasites (GAPs) that arrest in the liver and induce sterile immunity leads to the upregulation of miR-155 levels in the liver (**Figure 1b**), particularly in non-parenchymal cells including liver-resident macrophages, or Kupffer cells (Hentzschel et al., 2014). Additional studies are warranted to investigate plasma miRNA profiles specifically associated with this phase, which could be used in RT-qPCR-based assays for the early identification of silent infections.

## And Seek: The Symptomatic Erythrocytic Phase

After release from infected hepatocytes, merozoites invade erythrocytes and mature from ring stages to trophozoites, and ultimately, to schizonts (**Figure 1c**). The burst of iEs to release daughter merozoites results in exponential growth of the parasite population, and some of these merozoites develop into sexual forms, or gametocytes. The clinical symptoms of malaria are mainly attributed to the rupture of schizont stage-iEs and the subsequent release of parasite-derived toxins, which stimulate innate immune cells to produce cytokines and inflammatory mediators causing periodic episodes of febrile illness (Oakley et al., 2011; Gazzinelli et al., 2014). Circulating cytokines also induce the upregulation of adhesion molecules by endothelial cells which, in turn, increase parasite sequestration (Gazzinelli et al., 2014). This phenomenon is mediated by *Pf* erythrocyte membrane protein 1 (PfEMP1), a family of proteins present on the surface of iEs and encoded by approximately 60 *var* genes (Hviid and Jensen, 2015). PfEMP-1 can bind to several host receptors on the surface of capillary endothelium, uninfected erythrocytes, placental syncytiotrophoblasts, and platelets (Rowe et al., 2009; Turner et al., 2013; Wassmer et al., 2015). Such receptors include intercellular adhesion molecule-1 (ICAM-1), CD36, endothelial protein C receptor (EPCR), gC1qR, chondroitin sulfate A (CSA), or complement receptor 1 (Jensen et al., 2020). By binding to the vessel walls, mature forms of *Pf* remove themselves from the circulation, thereby avoiding clearance by the spleen (Cranston et al., 1984; Buffet et al., 2011). However, the local accumulation of iEs disrupts or completely abrogates the blood flow, promotes clotting, triggers endothelial cell and blood-brain barrier disruption, potentially leading to the extravasation of vascular content in the parenchymal tissue and increased local inflammation. These mechanisms have all been linked to SM (Gazzinelli et al., 2014).

Cell-cell interactions, as well as the immune response of the host are highly likely to influence the expression of miRNAs during malaria infection. Remarkably, only human miRNAs are found in *Pf*-iEs (Rathjen et al., 2006; Xue et al., 2008), and it has been reported that human erythrocytes miRNAs could translocate and integrate into the parasite messenger RNAs to block their translation (LaMonte et al., 2012). In particular, miR-451 and let-7i were found abundant in sickle erythrocytes, and together with miR-223, reduced parasite growth (LaMonte et al., 2012).

A study using controlled human *Pf*-infection in adults identified a profile of 84 miRNAs associated with T cell and B cell activation, indicating a pivotal role of miRNAs in inter-individual variability in the immune response to falciparum malaria. Volunteers with increased levels of miR-15a-5p, miR-30c-5p, and miR-30e-5p had a higher frequency of activated and proliferating T cells and could control their *Pf* parasitemia more effectively after infection (Burel et al., 2017).

Similarly, host genetics can regulate *Pf* infection (Gupta et al., 2013; Gupta et al., 2015b; Gupta et al., 2017a), and the protective effect of rs114136945 minor allele on parasitemia mediated through miR-598-3p expression was recently reported (Dieng et al., 2020). In a study carried out in Thailand, the downregulation of both miR-451 and miR-16 was reported in 19 malaria patients compared to healthy individuals (Chamnanchanunt et al., 2015). Subsequent work on a smaller number of participants found 8 differentially expressed miRNAs between *Pf* malaria patients and healthy controls (Bertrams et al., 2021).

### Severe Malaria: Multi-Organ and Physiological Dysfunctions

Severe *Pf* malaria is characterised by the sequestration of iEs in the microvasculature of the host (Miller et al., 2002). Combined with an exacerbated production of inflammatory mediators, this accumulation leads to the dysfunction of peripheral organs, either alone or in combination, leading to severe complications. These include acute respiratory distress syndrome, (ARDS, affecting the lungs), jaundice (liver), acute kidney injury (AKI, kidneys) or cerebral malaria (CM, brain) (White et al., 2013; Milner et al., 2014). The hard WHO cut-offs for these severe complications at the time of admission exclude patients with parameters just above or below the defined threshold. Thus, determining direct or indirect markers of early and still progressing organ dysfunction in the context of SM could revolutionize its clinical management. A recent, large-scale study using a combination of *in vitro* models and plasma samples from Mozambican children infected with *Pf* and diagnosed with SM or UM demonstrated for the first time the association between both hsa-miR-3158-3p and hsa-miR-4497 with SM, its complications, and *Pf* biomass (**Figure 1c**) (Gupta et al., 2021a). These findings suggest that although unable to produce miRNAs (Xue et al., 2008), *Plasmodium* may manipulate the production of host miRNAs. In turn, these changes in small molecule levels have the potential to not only shed light on the molecular mechanisms involved in the pathogenesis of SM, but also be used as signatures associated with individual complications. As SM is a broad spectrum disease, such miRNA signatures may help identify complications and tailor treatment approaches to increase survival.

#### Neurological Changes: Cerebral Malaria (CM)

CM is an acute neurological complication and often lethal form of SM. It has a fatality rate up to 30% in treated patients (Wassmer et al., 2015), and neurocognitive sequelae are frequent in survivors (WHO, 2014). CM cases are predominantly seen in African children under five, as the high malaria transmission intensity in sub-Saharan Africa leads to the development antimalarial immunity during childhood. However, in South East Asia, where malaria transmission is seasonal and not intense enough to induce robust immunity, CM cases are mainly found in older children and adults (Sahu et al., 2015). In addition, while CM is mainly accompanied by severe anemia and/or metabolic acidosis in African children, it is very often reported in combination with other organ involvement such as lungs, liver and kidneys leading to ARDS, jaundice and AKI, respectively in Asian adults (Wassmer et al., 2015). The mechanisms behind these distinct clinical features between African children and South East Asian adults is still poorly understood.

In addition, CM is challenging to diagnose using the tools currently available. An autopsy study in Malawi demonstrated that up to 23% of children clinically diagnosed with CM had, in fact, another cause of death based on post-mortem examination (Taylor et al., 2004). Further work reported that CM was indeed overly misdiagnosed when assessed by parasite load alone; 38% *versus* only 1% who truly fulfilled WHO criteria (Makani et al., 2003). Over the past decade, neuroimaging studies in endemic areas have improved our understanding of CM (Mohanty et al., 2014). Distinct age-dependent brain changes identified by magnetic resonance imaging (MRI) on admission were recently linked to poor outcomes. In pediatric CM, severe brain swelling with brain stem herniation was associated with fatality (Seydel et al., 2015), a feature not observed in fatal adult cases (Mohanty et al., 2011; Maude et al., 2014). Our team demonstrated that in the latter group, global cerebral hypoxic injury was associated with mortality (Sahu et al., 2020). While the early identification of such features may prove critical to inform clinical management and improve survival in patients at risk, access to MRI facilities remains extremely limited in malaria endemic countries due to operational, logistical and financial challenges (Latourette et al., 2011). In addition, the occurrence of specific pathogenic features in different age groups may be difficult to detect and differentiate in adolescents or young adults. Therefore, alternative biomarkers of brain changes identified by MRI and validated in both age groups would represent a powerful prognosis tool to identify patients at risk of developing fatal disease, inform clinical management, and decrease mortality in CM. In view of these challenges, miRNAs found associated with defined MRI features could be helpful in CM diagnosis.

miR-155 was found to be an important player in the CM pathogenesis *via* negative regulation of blood-brain-barrier integrity and T cell function (Barker et al., 2017). In addition to miR-155, murine studies have yielded a series of biomarker candidates for CM, including miR-19a-3p, miR-540-5p, miR-223-3p, miR-142-3p, miR-19b-3p, let-7i, miR-27a, miR-150, miR-146a, miR-193b, miR-205, miR-215 and miR-467a (El-Assaad et al., 2011; Cohen et al., 2018; Martin-Alonso et al., 2018). These miRNAs are significantly involved in several pathways relevant to CM, including TGF-β, inflammation, TNF signaling, monocyte sequestration in cerebral microvessels, endocytosis (El-Assaad et al., 2011; Cohen et al., 2018; Martin-Alonso et al., 2018). Among the miRNA candidates identified, miR-146a-5p and miR-150-5p were also associated with CM in Indian patients (Gupta et al., 2021b), together with miR-222-3p and miR-3158-3p (**Figure 1c**). Upon further analysis, high miR-150-5p and miR-3158-3p levels were associated with fatal CM. miR-3158-3p levels decreased significantly in CM survivors at day 30 post-treatment, strongly suggesting the CM specificity of this miRNA. Lastly, miR-3158-3p levels were found correlated with hypoxia in the brain of adults, and negatively correlated with increased brain volume of children, both identified by MRI (Gupta et al., 2021b). This indicates that the production of miR-3158-3p is decreased in CM patients with high brain volume on admission, a feature associated with a poor outcome in children (Seydel et al., 2015; Sahu et al., 2020). Inversely, miR-3158-3p levels increased in patients with high hypoxia on admission, a hallmark of fatal adult CM (Sahu et al., 2020). While further validation of the association between miR-3158-3p levels and MRI features of poor outcomes in CM is needed, our findings support the potential use of miR-3158-3p for CM prognosis in children and adults in lieu of neuroimaging (Gupta et al., 2021b).

#### Liver Dysfunction

Several studies using the murine model of *P. chabaudi* infection identified differentially expressed miRNAs in the liver of infected animals (Delic et al., 2011; Al-Quraishy et al., 2012; Dkhil et al., 2016). An upregulation of three and a downregulation of 16 miRNAs was reported in the mouse liver following infection. Remarkably, miRNA expression pattern persisted in immune mice even after re-infection, suggesting that the development of a protective immunity against *P. chabaudi* infection may be regulated in part by miRNAs (Delic et al., 2011). Another study reported 169 miRNAs downregulated in liver tissue obtained from *P. chabaudi*-infected mice, while miR-142-5p and miR-342-3p were found upregulated (**Figure 1c**) (Al-Quraishy et al., 2012). Similarly, a downregulation of 18 and upregulation of 14 miRNAs were reported in the liver during the acute phase of *P. chabaudi* infection (Dkhil et al., 2016).

#### Placenta: Pregnancy-Associated Malaria (PAM)

Pregnancy-associated malaria (PAM) is defined by the sequestration of iEs in the placental intervillous spaces. This sequestration is mediated by binding of VAR2CSA (Salanti et al., 2003) to chondroitin sulfate A (CSA) expressed at the surface of the placental syncytiotrophoblast (Zakama et al.; Fried and Duffy, 1996), contributing to inflammatory infiltrates and reduced nutrient transfer to the fetus (Rogerson et al., 2018), ultimately causing adverse outcomes including low birth weight, preterm birth, stillbirth, and miscarriage (Nosten et al., 2004). In addition, women who are pregnant for the first time generally lack immunity to antigenic variant presented by *Pf* parasites that accumulate selectively in the placenta, which put them at a higher risk of infection compared to non-pregnant women (Brabin et al., 2004; Gamain et al., 2007). The risk of infection decreases in the second trimester compared to first trimester in primigravid women. The infection risk also decreases after successive pregnancies (Gamain et al., 2007). Similarly to other organ-specific dysfunctions induced by *Pf*, increased levels of miR-517c (**Figure 1c**), an immunomodulator in pregnancy and tumorigenesis (Bullerdiek and Flor, 2012), were reported in mothers with PAM when compared to non-infected controls (Moro et al., 2016). miR-146a rs2910164 polymorphism was found to increases the odds of PAM occurrence in primigravid Ghanaian women, suggesting a role for miR-146a in this complication (van Loon et al., 2019). The authors of the study suggested that miR-146a is involved in protective malarial immunity, particularly its innate component.

#### Other Organ and Physiological Dysfunctions

A limited number of studies have been carried out to identify miRNAs associated with SM complications and organ dysfunction such as acidosis, severe anemia, ARDS, or AKI. Higher levels of miR-3158–3p were measured in Mozambican children who had acidosis or ARDS compared to UM children. In contrast, miR-4497 was associated with severe anemia and ARDS (**Figure 1c**) (Gupta et al., 2021a). A review suggested that miR-210; miR-125b and miR-181b; as well as miR 17–92 and miR-24 could be promising miRNA-based biomarkers of acidosis, ARDS and renal failure, respectively (**Figure 1c**) (Chamnanchanunt et al., 2017). However, miR-210-3p was not found associated with SM in a recent study conducted in India (Gupta et al., 2021b). Because this is still an emerging field, the data on biomarkers of malaria-associated organ dysfunction and severe complications remain limited. The resultant knowledge gap needs to be addressed to identify miRNAs associated with ARDS, AKI, acidosis and SA (**Figure 1c**). In turn, these tools will help researchers and clinicians to *i)* increase the granularity of the SM diagnosis and *ii)* determine pathogenetic pathways and potential therapeutic targets in SM (Wassmer et al., 2015). It is also noteworthy that grouping patients with single distinct SM complications is challenging. Indeed, acidosis, for example, is caused by the host anaerobic glycolysis due to tissue hypoxia following iE sequestration (Rubio et al., 2016) and develop in combination with other complications in most patients. Therefore, global or combination profiling may be an easier approach than identifying the profiles of miRNAs associated with specific dysfunction.

Thrombocytopenia is common in malaria (Lacerda et al., 2011; Anvikar et al., 2020) and has been associated with SM (Anvikar et al., 2020). The miRNA pair miR-4454/miR-7975 was found associated with low platelet counts (Santos et al., 2021). In addition, the roles of extracellular vesicles (EVs), small membrane-bound vesicles that can be classified based on their size, origin, and functions (EL Andaloussi et al., 2013), have recently emerged in the different stages of malaria life cycle, as well as in the pathogenesis of SM (Babatunde et al., 2020; Cheng et al., 2020). A high number of EVs can be found during pathological conditions (Combes et al., 2004; Nantakomol et al., 2011), and they can act as immunomodulators during *Plasmodium* infection either directly (Mantel and Marti, 2014) or *via* their miRNA cargo (Mantel et al., 2016; Wang et al., 2017). Indeed, EV contain miRNAs with regulatory functions, and those are protected from degradation by RNases (Cheng et al., 2014; Vojtech et al., 2014). A study reported that EV-derived miR-150-5p, miR-15b-5p and let-7a-5p were significantly up regulated in malaria patients compared to healthy individuals (Ketprasit et al., 2020). let-7a-5p miRNA was associated with falciparum malaria (Ketprasit et al., 2020), and miR-150-5p, miR-15b-5p and let-7a-5p with *Pv* infection (Ketprasit et al., 2020).

### Chronic Malaria

In high transmission settings, intense exposure to malaria drives the development of anti-disease immunity, whereby individuals control their immune response to infection, ultimately resulting in asymptomatic malaria (AM) (Kimenyi et al., 2019). By definition, it is characterised by the lack of apparent clinical symptoms, and infected individuals do not seek treatment. They are missed by passive surveillance while remaining an important gametocytes reservoir, and such infections contribute to the persistence of malaria transmission (Galatas et al., 2016; Andolina et al., 2021). AM has been perceived as relatively benign until a study demonstrated that it was associated with recurrent episodes of symptomatic parasitemia, chronic anemia, maternal and neonatal mortality, co-infection with invasive bacterial disease, cognitive impairment, and continuous transmission (Chen et al., 2016). The authors proposed to rename the misleading AM to chronic malaria (Chen et al., 2016).

As a large proportion of AM infections are associated with low-density parasitemia, these individuals are also likely to be missed by conventional tests such as microscopy and rapid diagnostic tests, which have limited detection sensitivity (Okell et al., 2012; Bousema et al., 2014). New approaches are therefore needed for large scale populations screening. miR-3158-3p and miR-4497 were found associated with parasite biomass (Gupta et al., 2021a) so it could be postulated that these miRNAs are promising candidate to identify sub-patent infections. However, further investigations are warranted to determine the parasitemia detection threshold afforded by these miRNAs.

In a similar way, *Pv* has also been assumed to be unharmful due to relatively low parasitemia levels and high proportions of AM cases. However, evidence is mounting that vivax malaria is debilitating and potentially causes life-threatening complications similar to the ones reported in *Pf* infection (Gupta et al., 2015a; Gupta et al., 2016a; Anvikar et al., 2020). miR-451 and miR-16 levels were reported downregulated in *Pv* patients compared to controls (**Figure 1c**) (Chamnanchanunt et al., 2015). The miRNA pair miR-4454/miR-7975 was upregulated while miR-520f-3p, miR-150-5p and let-7b-5p were down regulated in patients with *Pv* infection (Santos et al., 2021). The miR-150-5p association with *Pv* infection has not been consistent in Thai and Brazilian populations (Ketprasit et al., 2020; Santos et al., 2021), suggesting the need for further validation. Another study found miR-7977, miR-28-3p, miR-378-5p, miR-194-5p and miR-3667-5p associated with *Pv* infection, and the authors postulated that miR-7977 exacerbated its pathology *via* the UBA52 or TGF-beta signalling pathways (Kaur et al., 2018). These results suggest that host miRNA levels are also influenced by the presence of *Pv* infection, which opens new avenues to identify miRNA-based biomarkers that can not only differentiate between malaria patients from healthy individuals but could also identify different *Plasmodium* species. To the best of our knowledge, this research field remains limited and only miR-451 levels were found differentially expressed between patients infected with *Pf* (n=3) and *Pv* (n=16) (Chamnanchanunt et al., 2015). However, the sample size for this comparison was small. Combined *Plasmodium* species-specific miRNA-based biomarkers could play an important in the context of malaria elimination programs to detect multi-species infections. Currently, these programs are mainly focusing on *Pf*, which can lead to the rise of other *Plasmodium* species.

Recent work demonstrated for the first time a large parasite biomass in the spleen of AM individuals compared to the peripheral circulation, which is likely to contribute to anemia (Kho et al., 2021a; Kho et al., 2021b). Both reticulocytes and asexual *Pv* parasites were observed in the splenic tissue, suggesting that the measure of the peripheral parasitemia alone vastly underestimates the *Pv* biomass in AM (Kho et al., 2021a). miR-4497 may play an important role in detecting hidden splenic parasites in AM individuals, as it was found associated with parasite biomass and SA (Gupta et al., 2021a). In addition, another study reported the downregulation of 25 miRNAs in the spleen obtained from mice infected with *P. chabaudi* (Al-Quraishy et al., 2012). However, additional validation work is needed to identify miRNA-based biomarkers with a potential to detect splenic parasites in individuals with AM. This approach will be crucial to identify and treat such cases in order to interrupt malaria transmission.

A hallmark of *Pv* is its ability to form dormant liver stages, or hypnozoites. These can reactivate, causing recurrent episodes of malaria (Dayananda et al., 2018). However, frequent episodes do not allow patients to recover from hematological damage, leading to severe anemia (Tjitra et al., 2008). Because current diagnostic tools cannot detect hypnozoites, a 14-day course of primaquine is recommended by the WHO for all patients who are not deficient in the glucose-6-phosphate dehydrogenase (G6PD) enzyme (WHO, 2015). In view of the challenges associated with the wide implementation of a long treatment course and practical pretreatment testing to identify G6PD-deficient individuals at risk of severe hemolysis (Baird et al., 2016), a screening tool to detect these hypnozoites may be key to reduce these bottlenecks. miRNA-based assays could fill this gap and support current elimination efforts.

Overall, miRNAs found associated with the symptomatic phase of the malaria life cycle in different studies, specifically miR-146a-5p, miR-150-5p, miR-3158-3p and miR-4497 have the potential to develop diagnostic and prognostic tools to identify malaria patients, organ dysfunction, and patients at the risk of developing SM associated life-threating complications, inform their clinical management, and decrease SM associated mortality.

## Ready for Take off: Gametocytes

Gametocytes are sexual forms of the malaria parasite, which enable the establishment of infection in mosquitoes from its mammalian host, a crucial step in malaria transmission (Bousema and Drakeley, 2011). Similarly to mature asexual parasites in iEs, it has been postulated that immature gametocytes are “sequestered” away from the peripheral blood and the host immune cells. This process would ensure their safe maturation before they are released into the circulation (Bousema and Drakeley, 2011). Indeed, a high prevalence and abundance of early *Pf* sexual stages was identified in the bone marrow (BM), which was linked to both dyserythropoiesis and severe anemia (Aguilar et al., 2014). It is noteworthy that the presence of gametocytes in the BM leads to transcriptional changes of miRNAs expression involved in erythropoiesis. miR-221/222, miR-24 and miR-191 levels were all decreased in the BM during *Pv* infection, and returned to normal during convalescence (**Figure 1c**). In contrast, miR-144 and miR-150 levels were both upregulated during infection (**Figure 1c**) (Baro et al., 2017). However, these findings need further validation using *in vitro* models validated with clinical samples. Indeed, an *in vitro* model of tridimensional co-culture in a Matrigel scaffold with *Pf* gametocytes and self-assembling spheroids of human bone marrow mesenchymal cells (hBM-MSCs) was recently described (Messina et al., 2018), where the immature gametocytes adhered to hBM-MSCs *via* trypsin-sensitive parasite ligands exposed on the erythrocyte surface (Messina et al., 2018). This model could be used to identify altered miRNA expression by comparing the models with and without gametocytes, prior to validating these findings in infected individuals.

## Other Relevant Considerations

### Age, Immunity, and miRNAs

Falciparum malaria-associated mortality is higher in children under five years of age, who lack immunity to the parasite (Streatfield et al., 2014). In contrast, adults in endemic areas have been exposed regularly, and have progressively built up an anti-disease immunity leading to sub-clinical infections (Schwartz et al., 2001). Because the development of such immunity depends on malaria exposure, SM cases are more often seen in African children as high malaria transmission intensity in sub-Saharan Africa leads to develop antimalarial immunity during childhood. However, in South East Asia, where malaria transmission is not sufficiently intense to induce robust immunity, SM mainly affects older children and adults (Sahu et al., 2015). Therefore, biomarkers of malaria infection validated in both age groups and transmission intensities would represent a powerful prognosis tool to identify patients at risk of SM fatality, inform their clinical management, and decrease SM associated mortality. In the context of miRNA-based biomarkers, miR-3158-3p is the only candidate that has been associated with SM and subcategories of SM in both Mozambican children (Gupta et al., 2021a), as well as Indian adults and children (Gupta et al., 2021b). Further validations and translational efforts are now needed, and the former may also help elucidating the mechanisms behind distinct the different clinical profiles of SM found in African children and South East Asian adults (Wassmer et al., 2015).

### Technical Considerations

Studies investigating the potential diagnostic benefit of miRNA-based biomarkers in SM all used samples obtained patients in malaria endemic countries where co-infections are frequent. Because the symptoms of malaria infection are non-specific, they can easily overlap with concurrent infections, and one important consideration for future studies is to ensure that miRNAs candidates have been tested against non-malarial diseases. Indeed, the observed miRNA level changes in malaria patients can be influenced by inflammation due to the presence of other pathogens and samples obtained from patients with non-malarial diseases should be used as additional controls. There are several techniques available for the identification of miRNAs (recently reviewed in (Sempere et al.; Condrat et al., 2020) including classical and low-throughput methods such as northern blot and RNA protection assays, requiring large quantities of total RNA, and advanced methods such as microarray, nCounter Nanostring technology, NGS and reverse transcription quantitative PCR (RT-qPCR). Among these, microarray, nCounter Nanostring technology and NGS are usually used in the discovery phase or initial screening of the study, while RT-qPCR assays are used in the validation phase (Sempere et al.; Condrat et al., 2020). However, the use of RT-qPCRs can be limited due to the lack of appropriate normalizing controls. In our opinion, endogenous miRNA controls (ECs) should be disease-specific as ECs with stable expression in the case and control groups found suitable for cancer studies may not be appropriate for malaria samples. A study reported the combination of hsa-miR-30d-5p and hsa-miR-191–5p as the suitable ECs to normalize the miRNA RT-qPCRs data obtained using plasma from patients with malaria infection (Gupta et al., 2021a). The combination of ECs had a 0.044 NormFinder stability value. In addition, no differences were found in Ct values of the two ECs when compared between Mozambican patients with SM and UM (Gupta et al., 2021a), further confirming their stable expression in different malaria pathologies.

### Future Directions

Lateral flow assays (LFA) developed to detect circulating miRNAs associated with different type of cancers are showing promising results. A gold nanoparticle-based LFA was able to detect a minimum concentration of 60 pM of miR-215 within 20 minutes in aqueous solutions and biological samples (Gao et al., 2014). Similarly, another LFA was able to detect miR-21, miR-155 and miR-210 with detection limits of 0.073, 0.061 and 0.085 nM, respectively (Zheng et al., 2018). Lastly a Gold@Silica nanocomposite-labeled LFA allowed the visual detection of miR-21 in cancer cells and human serum down to 1 pM (Dong et al., 2021). While these concentration thresholds are highly likely to afford the detection of candidates such as miR-3158-3p in SM, additional studies using plasma from different groups of infected individuals are needed to assess the sensitivity and reliability of such LFAs in malaria diagnosis. They could represent a game changer in the field, especially when the number of *pfhrp2* deletion reports are increasing (Gupta et al., 2017b; Gendrot et al., 2019; Galatas et al., 2020). PfHRP2-based rapid diagnostic tests (RDTs) are the most widely used diagnostic method across malaria endemic countries (Gendrot et al., 2019). The advantage of miRNA-based LFA is that they could be designed to detect multiple miRNAs (Zheng et al., 2018). This would not only further enhance the sensitivity and specificity of the assay, but also allow a higher granularity in clinical diagnosis.

In addition to their potential as diagnostic tools, miRNA may also open new therapeutic avenues in SM, by either mimicking or inhibiting specific miRNAs associated with its pathogenesis. In the former case, miRNA mimics aim to restore the expression of miRNA that was lost. Inversely, anti-miRNAs (antimiRs) are single stranded oligonucleotides, which are chemically designed to block the function of miRNA candidate overexpressed during the course of the disease (Rupaimoole and Slack, 2017). Attempts have been made using miRNA mimics and antimiRs for the therapeutic intervention in other diseases, including a mimic of the tumour suppressor miRNA miR-34, which reached phase I clinical trials for treating cancer, and antimiRs targeted at miR-122, which reached phase II trials for treating hepatitis (Rupaimoole and Slack, 2017; Hanna et al., 2019). Similarly, miR-16, miR-21, miR-29, miR-92 and miR-155 based therapeutics are also in phase I and II trials to test their efficacy to cure wound healing, heart failure, cancer and other diseases (Hanna et al., 2019). While these approaches are not currently explored in malaria research, further investigations in the role of miR-3158-3p in regulating the genes associated with brain injury and processes relevant to SM may be promising. In addition, the WHO recently endorsed the RTS,S/AS01 (Mosquitix™) malaria vaccine for use among children in sub-Saharan Africa and in other regions with moderate to high falciparum malaria transmission. Several groups reported that vaccines could influence serum miRNA levels: a study demonstrated a change in miRNA expression levels in samples obtained from UK children who received vaccination against influenza (H1N1) (Drury et al., 2019). Using mice models, another study showed that vaccines associated with or without protection against respiratory syncytial virus led to different circulating miRNA profiles (Atherton et al., 2019). The roll-out of the Mosquitix™ vaccine will represent a great opportunity to investigate over-expressed or down-regulated miRNAs in protected vaccinated individuals. In turn, these could inform miRNA-based therapeutics against *Pf* infection.

## Conclusions

Microscopy remains the gold standard for malaria diagnosis. However, no currently available methods can identify patients with parasite sequestration-associated tissue injury or predict their level of infection severity. There is a need for new tools and technologies easily implementable in malarious areas to improve diagnosis and increase survival. miRNAs, which are rapidly released in biofluids upon infection and organ damage, could serve as a measure of tissue injury and be used to accurately detect parasites not only in infected humans but also in mosquitoes, to contribute to malaria elimination efforts.

## Author Contributions

HG and SW designed and conceptualized the manuscript. HG carried out the literature search, and together with SW generated the first draft of the manuscript. Both authors reviewed and approved the final manuscript.

## Funding

This work was supported by the National Institute of Allergy and Infectious Diseases of the National Institutes of Health under Award Numbers U19AI089676 and R21AI142472, and by the Medical Research Council, UK, under Award Number MR/S009450/1. The content is solely the responsibility of the authors and does not necessarily represent the official views of the funders.

## Conflict of Interest

The authors declare that the research was conducted in the absence of any commercial or financial relationships that could be construed as a potential conflict of interest.

## Publisher’s Note

All claims expressed in this article are solely those of the authors and do not necessarily represent those of their affiliated organizations, or those of the publisher, the editors and the reviewers. Any product that may be evaluated in this article, or claim that may be made by its manufacturer, is not guaranteed or endorsed by the publisher.
